# Mucin Hypersecreting Intraductal Papillary Neoplasm of the Pancreas

**DOI:** 10.1155/1999/81649

**Published:** 1999-04

**Authors:** Anthony W. Kim, Anna Ryan, Keith W. Millikan, Alexander Doolas

**Affiliations:** 1 Rush Medical College Rush Presbyterian-St. Luke's Medical Center Chicago IL 60612 USA; 2 Department of Pathology Rush Presbyterian-St. Luke's Medical Center Chicago IL 60611 USA; 3 Department of General Surgery Rush Presbyterian-St. Luke's Medical Center Chicago IL 60612 USA; 4 University Surgeons S.C. Suite 810 Professional Building Rush Presbyterian-St. Luke's Medical Center 1725 W. Harrison St. Chicago IL 60612 USA

## Abstract

Mucin Hypersecreting Intraductal Papillary Neoplasm is a rare neoplasm that arises from ductal epithelial cells. This entity is distinct from the more commonly known Mucinous Cystadenoma or Mucinous Cystadenocarcinoma. Despite this distinction, it has been erroneously categorized with these more common cystic neoplasms. Characteristic clinical presentation, radiographic, and endoscopic findings help distinguish this neoplasm from the cystadenomas and cystadenocarcinomas. Histopathologic identification is not crucial to the preoperative diagnosis. This neoplasm is considered to represent a premalignant condition and, therefore, surgical resection is warranted. Prognosis, following resection, is felt to be curative for the majority of patients.

We present two cases of Mucin Hypersecreting Intraductal Papillary Neoplasm and discuss their diagnosis and surgical therapy.


*HPB Surgery*, 1999, Vol. 11, pp. 175–184
Reprints available directly from the publisher
Photocopying permitted by license only

© 1999 OPA (Overseas Publishers Association) N.V.
Published by license under
the Harwood Academic Publishers imprint,
part of The Gordon and Breach Publishing Group.
Printed in Malaysia.

*Case Report*

### Mucin Hypersecreting Intraductal Papillary Neoplasm of the Pancreas

ANTHONY W. KIM [a], ANNA RYAN [b], KEITH W. MILLIKAN [c,*] and ALEXANDER DOOLAS [c]

[a] *Rush Medical College, Rush Presbyterian-St. Luke's Medical Center, Chicago, IL 60612;*
[b] *Department of Pathology, Rush Presbyterian-St. Luke's Medical Center, Chicago, IL 60611;*
[c] *Department of General Surgery, Rush Presbyterian-St. Luke's Medical Center, Chicago, IL 60612*

*(Received 24 October 1997; In final form 12 April 1998)*

**Mucin Hypersecreting Intraductal Papillary Neoplasm is a rare neoplasm that arises from ductal epithelial cells. This entity is distinct from the more commonly known Mucinous Cystadenoma or Mucinous Cystadenocarcinoma. Despite this distinction, it has been erroneously categorized with these more common cystic neoplasms. Characteristic clinical presentation, radiographic, and endoscopic findings help distinguish this neoplasm from the cystadenomas and cystadenocarcinomas. Histopathologic identification is not crucial to the preoperative diagnosis. This neoplasm is considered to represent a premalignant condition and, therefore, surgical resection is warranted. Prognosis, following resection, is felt to be curative for the majority of patients.**

**We present two cases of Mucin Hypersecreting Intraductal Papillary Neoplasm and discuss their diagnosis and surgical therapy.**

*Keywords:* Mucin Hypersecreting Intraductal Papillary Neoplasm, cystic neoplasm, pancreas

### INTRODUCTION

Mucin Hypersecreting Intraductal Papillary Neoplasm is a distinct entity from the more commonly known Mucinous Cystadenoma or Mucinous Cystadenocarcinoma. It has been referred to by many other names including mucinous duct ectasia, intraductal papillary neoplasm or adenocarcinoma, ductectatic-type or variant of mucinous cystadenoma or mucinous cystadenocarcinoma, multiple primitive endoluminal tumors of the main pancreatic duct of Wirsung, diffuse villous carcinoma of the duct of Wirsung, carcinoma *in situ* of the pancreas, and intraductal mucin hypersecreting neoplasms or carcinomas [1–6]. This neoplasm is characterized by dilatation of the main pancreatic duct of Wirsung and the secondary ducts with the production of mucin and by its malignant potential [1,3,5]. Because of this malignant potential, surgical therapy is war-

*Address for correspondence: University Surgeons S.C., Suite 810 Professional Building, Rush Presbyterian-St. Luke's Medical Center, 1725 W. Harrison St., Chicago, IL 60612.

ranted in appropriate patients. Unlike the disappointing results with surgery encountered with the more common adenocarcinomas of the pancreas, surgery for this neoplasm is associated with a very good outcome.

### CASE REPORTS

### Case 1

A 73-year old woman presented with a four year history of post-prandial abdominal pain. Interestingly, her older sister died recently from pancreatic cancer. Her mother was a diabetic and had ovarian cancer, another sister had colon cancer, and her brother had prostate cancer. The patient quit smoking 40 years ago and maintains minimal alcohol use. The patient presented to her primary physician with worsening of her symptoms in 1996 and associated five pound weight loss. An ultrasound of the gallbladder demonstrated no cholelithiasis, but there was lymphadenopathy in the perigastric region. Computed tomography (CT) showed a cystic mass within the pancreatic head measuring 6 cm in length and a 2 cm density along the inferior aspect of the pancreas (Fig. 1). An endoscopic retrograde cholangiopancreatography (ERCP) examination revealed mucin extruding from the ampulla and ductal dilatation with an irregular strictured segment near the junction of the head and the body (Fig. 2). Preoperative Carcinoembryonic Antigen (CEA) was found to be 1.5 ng/mL (Normal <2.5 ng/mL nonsmoker, <5.0 ng/mL smoker) and Carbohydrate Antigen 19-9 was 12 U/mL (Normal <31 U/mL). Liver enzymes were within normal limits. Amylase was elevated at 256 U/L and glucose was mildly elevated at 131 mg/dL. She underwent a pylorus preserving pancreaticoduodenectomy. On gross examination, mucin extruded from the ampulla and on cross section the pancreatic ducts were more distended with mucin into cyst-like spaces (Figs. 3a–b). Frozen section confirmed tumor free margins. The patient had an uneventful post-operative course and was doing well at six months follow up. The final pathology of the mass indicated a Mucin Hypersecreting Intraductal Papillary Neoplasm of the pancreas with

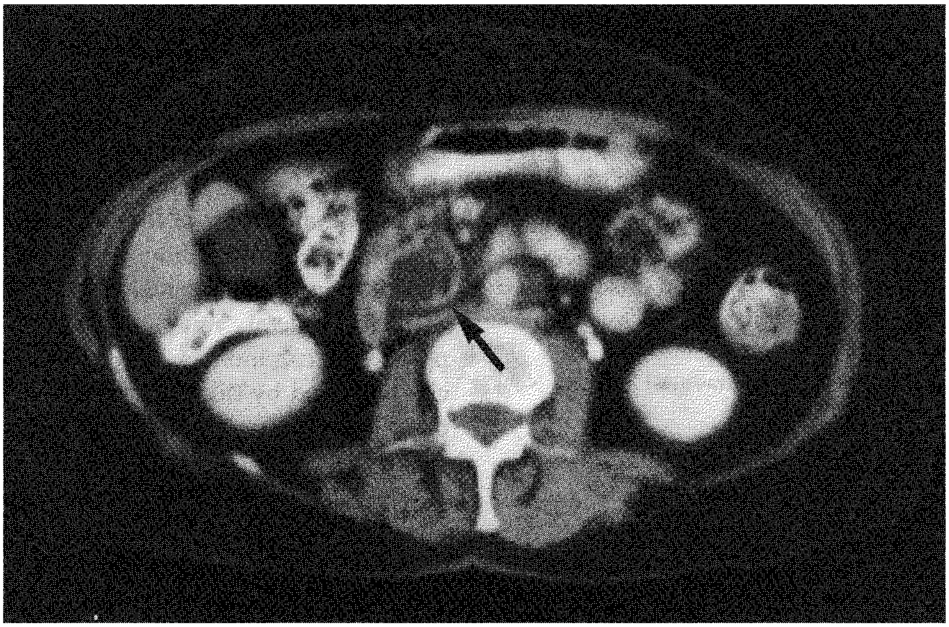

FIGURE 1 Case #1 CT of abdomen demonstrating a cystic mass within the pancreatic head (arrow).

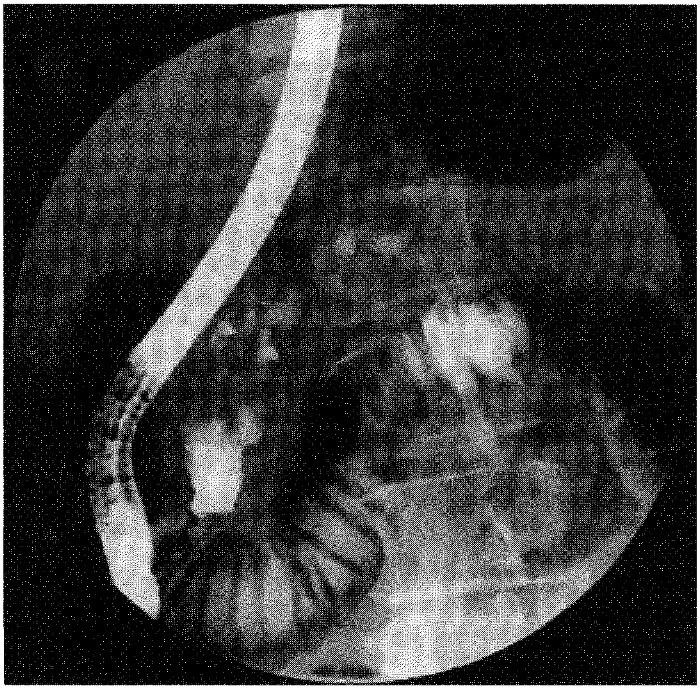

FIGURE 2 Case #1 ERCP demonstrating ductal dilatations with an irregular strictured segment near the junction of the head and the body.

a)

FIGURE 3 Case #1 Gross picture of Mucin Hypersecreting Intraductal Papillary Neoplasm. (a) Cross section of main duct with extrusion of viscous mucin (large arrow). Note absence of intraluminal masses and involvement of smaller branch ducts (small arrow). (b) Mucin extruding through dilated ampulla (arrow). (See Color Plate III).

b)

FIGURE 3 (Continued).

5% of the total area showing severe dysplasia or adenocarcinoma *in situ*. No invasion was seen. There was evidence of extravasation of mucin into the pancreatic stroma resulting in extensive sclerosis. There was also generalized periductal fibrosis and significant chronic pancreatitis. Margins were read as without atypia and the peripancreatic lymph nodes were free of tumor. Histologically, the duct of Wirsung and its branches showed ectasia and papillary hyperplasia of the epithelium along with luminal mucin (Figs. 4a–d).

### Case 2

A 76-year old woman presented with three episodes of pancreatitis, the last visit with an elevated Amylase (236 U/L) and Lipase (361 U/L). There was no abdominal pain, nausea, emesis, or weight loss. Previous medical history included hepatitis B virus infection, hypertension, and gastroesophageal reflux disease. Surgical history was significant for ovarian cyst removal, total abdominal hysterectomy, and a Marshal–Marchetti–Kranz procedure for urinary incontinence. There was either history of tobacco or ethanol use nor any significant family history. CT scan demonstrated diffuse pancreatic ductal dilatation but no masses or nodes (Fig. 5). An ERCP demonstrated a dilated major pancreatic duct with filling defects in the head of the pancreas (Fig. 6) and endoscopic examination revealed a patulous major papilla (Fig. 7). A sphincterotomy followed by brush cytology revealed benign cells. Measurements of CEA and CA 19-9 six months earlier were 6.9 ng/mL and $<8$ U/mL, respectively. One month later, CEA rose to 8.4 ng/mL while CA 19-9 remained at $<8$ U/mL. Liver enzymes were within normal limits. Pancreaticoduodenectomy was performed. Intraoperatively, the presumed involved region of the pancreas was removed and sent to pathology. The cut margin revealed papillary mucinous epithelium at the duct a)

b)

FIGURE 4 Case #1 Pathology Slides: (a) Low power view showing the lumen of a duct filled with papillary epithelium. (b) Papillae lined by tall columnar, mucin secreting cells with basally oriented nuclei. Goblet cells are seen. (c) Area of severe dysplasia (arrows). (d) Area of fibrotic pancreatic parenchyma (small arrow) with dissection of mucin (large arrow). (See Color Plate IV).

c)

d)

FIGURE 4 (Continued).

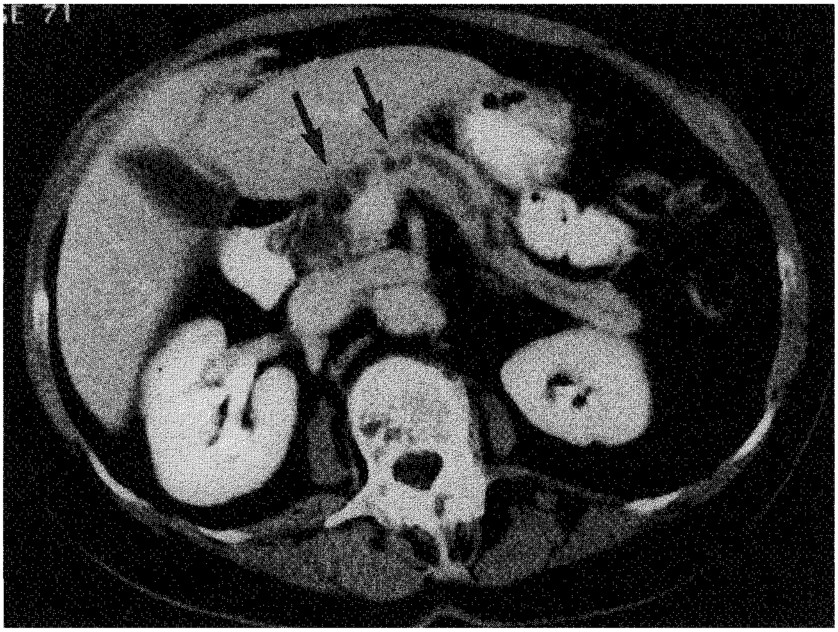

FIGURE 5 Case #2 CT of abdomen demonstrating diffuse pancreatic ductal dilatation, but no masses or lymphadenopathy (arrows).

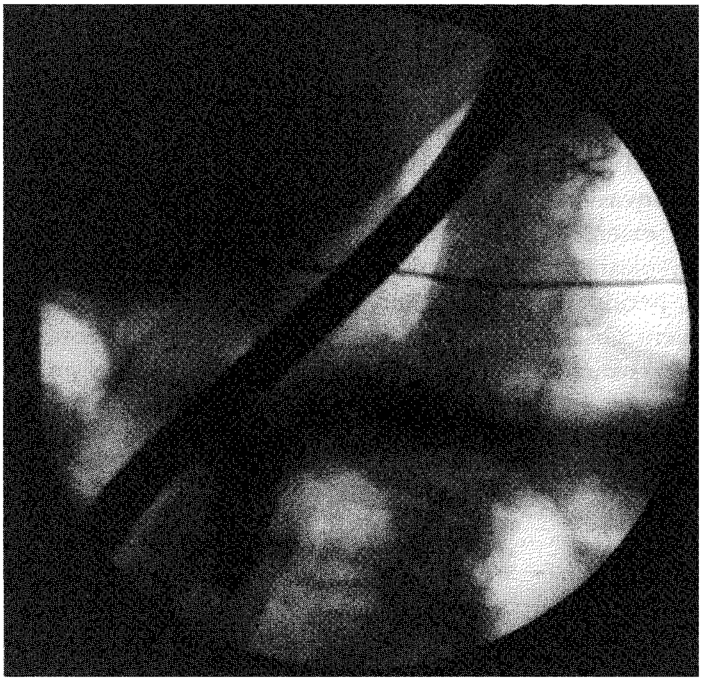

FIGURE 6 Case #2 ERCP showing a dilated major pancreatic duct with filling defects in the head of the pancreas.

FIGURE 7 Case #2 endoscopic picture demonstrating ERCP cannula placed through characteristic finding of a patulous papillae. (See Color Plate V).

margin necessitating a further 2.5 cm resection of the neck of the pancreas that revealed no further neoplasm. The postoperative course was unremarkable. Final pathology confirmed Mucin Hypersecreting Intraductal Papillary Neoplasm of the pancreas without invasion or lymph node involvement and with extensive duct ectasia. Foci of moderate dysplasia, papillary duct epithelial hyperplasia, periductal sclerosis and chronic pancreatitis were observed. The additional resected neck of the pancreas showed duct ectasia, periductal sclerosis, and focal chronic pancreatitis, but no dysplasia or neoplasm.

### DISCUSSION

Mucin hypersecreting intraductal neoplasms were first described as a separate neoplastic entity in 1982 [3,7,8]. The exact frequency of occurrence is unknown, although an estimation of 1% has been reported [9,10]. A review of the literature indicates uncertainty regarding a sex bias and age predilection [1,4,6]. The cystic nature of this entity has led to the mistaken classification of this tumor with the more common mucinous cystic tumors, Mucinous Cystadenoma and Mucinous Cystadenocarcinomas [1,3,9]. Although these neoplasms are considered cystic neoplasms they differ from the typical cystadenomas and cystadenocarcinomas by their clinical presentation and pathology. Epidemiologically, mucinous cystic tumors occur in younger patients with a predominance in females. Additionally, mucinous cystadenomas often originate in the tail whereas the Mucin Hypersecreting Intraductal Papillary Neoplasms tend to originate in the head.

The clinical presentation can include epigastric discomfort or severe pain sometimes associated with steatorrhea or weight loss [1,8]. Jaundice, cholangitis, and diabetes mellitus have also been described [1,7,8]. Obstructive jaundice may occur secondary to ductal mucin impaction or ampulla involvement when fistulas form into the bile duct or duodenum [1,7]. Increased amylase and lipase levels presumably are due to the pancreatitis that occurs in approximately 50–100% of the cases. These elevations are thought to be secondary to the mucin plugging of the ducts and the presence of intraluminal masses [3,4]. The pancreatitis may be acute or chronic and may result in exocrine and endocrine pancreatic insufficiency [1,3,5,6]. The finding of dilated ducts alone makes the distinction from chronic pancreatitis a difficult one [11]. Incidental discovery of this neoplasm in asymptomatic patients is another possible clinical presentation [1].

Grossly, the ectatic duct surfaces are either smooth and filled with mucin or contain papillary growths. Mucin within the ducts may be difficult to drain and can predispose the ducts to mucin plug formation that may ultimately lead to ductal dilation and pancreatitis [4]. In contrast, the Mucinous Cystadenomas and Cystadenocarcinomas have large dominant loculations of mucin surrounded by a fibrotic capsule [12] that, typically, do not communicate with the ductal system unless there is a fistulous formation between the two [9].

Histopathologically, the ducts are lined by a single layer of tall columnar cells [1, 3, 4, 8]. The surrounding pancreatic tissue can show perilobular and intralobular fibrosis [3]. This fibrosis is thought to be due to the leakage of the hypersecreted mucin into the pancreatic parenchyma [5]. Mild epithelial dysplasia constitutes the diagnosis of adenoma, whereas, severe dysplasia constitutes intraductal carcinoma (carcinoma *in situ*) [10]. The prevalence of carcinoma *in situ* or invasive lesions has been reported to be as high as 88% [6].

Diagnosis of Mucin Hypersecreting Intraductal Papillary Neoplasm may be suggested by ultrasound or CT when there is diffuse or segmented dilatation of the main duct or when there are intraluminal polypoid lesions. Confirmation is achieved by identifying characteristic endoscopic and pancreatographic findings [3,7,11]. The finding of a patulous papilla with drainage of frank mucin is highly suggestive of the diagnosis. Confirmation by ERCP shows a dilated Ampulla of Vater, or patulous papilla, with significant main pancreatic duct dilatation and intraluminal filling defects within the main duct and, occasionally, the side branch ducts [2–4,6–8,11,13]. The sensitivity, specificity, and accuracy of ERCP has been reported to be 91%, 91%, and 86% respectively [10]. Recently, pancreatoscopy has been advocated in diagnosing this neoplasm [3].

Imaging studies demonstrating increased tumor size has been shown to correlate with malignancy [2]. Although imaging studies cannot definitively distinguish among hyperplasia, adenoma, and adenocarcinoma, there have been criteria reported, based upon tumor size, that correlates with histopathologic tumor grade. ERCP studies suggest that ductal dilation greater than 8 mm in diameter have an increased incidence of being adenocarcinoma. Endoscopic ultrasound studies also suggest the same when cystic lesions are greater than 20 mm [13].

Recently, cytology has been emphasized for its greater diagnostic role. Compared to an endoscopic ultrasound and an ERCP, cytology was shown to have a higher sensitivity, specificity, and accuracy in detecting malignancy [13]. Surgery can be delayed in patients without cancerous cytology.

Overall, the prognosis of this neoplasm is very good and given the low malignant potential, resection, either by pancreaticoduodenectomy or distal pancreatectomy depending upon the site of the lesion, is recommended upon discovery. In a few cases, total pancreatectomy, despite its known sequelae, may be indicated [6,9]. The good prognosis with resection is yet another distinguishing characteristic of this neoplasm when compared to the other cystic neoplasms [1,4,8]. Lymph node involvement is usually not a factor, thus extensive nodal dissection is usually not indicated. Follow up does not include any special considerations as there have been no data showing recurrence or metastatic spread [1,3,4,6].

The findings in this case report were consistent both grossly and histologically with the previously described mucinous duct ectatic neoplasms. The patients in the two cases reported had components of the classic presentation of this neoplasm. Both had evidence of chronic pancreatitis with elevations in either amylase or lipase or both. Both patients had diagnostic imaging tests highly suggestive of Mucin Hypersecreting Intraductal Papillary Neoplasm and as a result underwent a pancreaticoduodenectomy. Gross examination of the tumor intraoperatively, in both cases, demonstrated expected changes and the pathologic diagnosis confirmed the clinical suspicions and imaging study findings. In both cases, the outcomes of the pancreaticoduodenectomy were uneventful and consistent with the good prognosis afforded by this rare neoplasm. Given the possibility of this neoplasm undergoing malignant transformation, surgical therapy was not only recommended, but also warranted in the two cases described.

### References

[1] Morohoshi, R., Kanda, M., Asanuma, K. and Kloppel, G. (1989). Intraductal Papillary Neoplasms of the Pancreas: A Clinicopathologic Study of Six Patients, *Cancer*, **64**, 1329–1335.

[2] Nagai, E., Ueki, T., Chijiiwa, K., Tanaka, M. and Tsuneyoshi, M. (1995). Intraductal Papillary Mucinous Neoplasms of the Pancreas Associated with So-called "Mucinous Ductal Ectasia": Histochemical and Immunohistochemical Analysis of 29 Cases, *Am. J. Surgical Pathology*, **19**(5), 576–589.

[3] Raijman, I., Kortan, P., Walden, D., Kandel, G., Marcon, N. E. and Haber, G. B. (1994). Mucinous Ductal Ectasia: Cholangiopancreatographic and Endoscopic Findings, *Endoscopy*, **26**, 303–307.

[4] Rickaert, F., Cremer, M., Deviere, J., Tavares, L., Lamibilliotte, J. P., Schroder, S., Wurbs, D. and Kloppel, G. (1991). Intraductal Mucin-Hypersecreting Neoplasms of the Pancreas: A Clinicopathologic Study of Eight Patients, *Gastroenterology*, **101**, 512–519.

[5] Santini, D., Campione, O., Salerno, A., Gullo, L., Mazzoleni, G., Leone, O., Martinelli, G. and Marrano, D. (1995). Intraductal Papillary-Mucinous Neoplasm of the Pancreas: A Clinicopathologic Entity, *Arch. Pathol. Lab. Med.*, **119**, 209–213.

[6] Rivera, J. A., Fernandez-del Castillo, C., Pins, M., Compton, C. C., Leewandrowski, K. B., Rattner, D. W. and Warshaw, A. L. (1997). Pancreatic Mucinous Ductal Ectasia and Intraductal Papillary Neoplasms: A Single Malignant Clincopathologic Entity, *Annals of Surgery*, **226**(6), 637–646.

[7] Itai, Y., Kokubo, T., Atomi, Y., Kuroda, A., Haraguchi, Y. and Terano, A. (1987). Mucin-Hypersecreting Carcinoma of the Pancreas, *Radiology*, **165**(1), 51–55.

[8] Kawarada, Y., Yano, T., Yamamoto, T., Yokoi, H., Imai, T., Ogura, Y. and Misumoto, R. (1992). Intraductal Mucin-Producing Tumors of the Pancreas, *Am. J. Gastroenterology*, **87**(5), 634–638.

[9] Warshaw, A. L. (1991). Mucinous Cystic Tumors and Mucinous Ductal Ectasia of the Pancreas, *Gastrointestinal Endoscopy*, **37**(2), 199–201.

[10] Solcia, E., Capella, C. and Kloppel, G. (1997). Atlas of Tumor Pathology, *Tumors of the Exocrine Pancreas*, **3**(20), 41–88.

[11] Gazelle, G. S., Mueller, P. R., Raafat, N., Halpern, E. F. and Warshaw, A. L. (1993). Cystic Neoplasms of the Pancreas: Evaluation with Endoscopic Retrograde Pancreatoscopy, *Radiology*, **188**, 633–636.

[12] Schoonbroodt, D., Zipf, A., Herrmann, G., Wenisch, H. and Jung, M. (1996). Pancreatoscopy and Diagnosis of Mucinous Neoplasms of the Pancreas, *Gastrointestinal Endoscopy*, **44**(4), 479–482.

[13] Uehara, H., Nakaizumi, A., Iishi, H., Tatsuta, M., Kitamra, T., Okuda, S., Ohigashi, H., Ishikawa, O., Takenaks, A. and Ishiguro, S. (1994). Cytologic Examination of Pancreatic Juice for Differential Diagnosis of Benign and Malignant Mucin-Producing Tumors of the Pancreas, *Cancer*, **74**(3), 826–833.

